# MIRA: A Transformer-Based Framework for Idler Roller Anomaly Detection and Localization

**DOI:** 10.3390/s25247469

**Published:** 2025-12-08

**Authors:** Younho Nam, Su Yeon Shim, Kyeong Su Shin, Young-Joo Suh

**Affiliations:** 1 Department of Computer Science and Engineering, Pohang University of Science and Technology, Pohang 37673, Republic of Korea; younho@postech.ac.kr; 2 Graduate School of Artificial Intelligence, Pohang University of Science and Technology, Pohang 37673, Republic of Korea; syshim@postech.ac.kr (S.Y.S.); ksshin@postech.ac.kr (K.S.S.)

**Keywords:** anomaly detection, belt conveyor idler, condition monitoring, transformer autoencoder, unsupervised learning

## Abstract

Monitoring the condition of belt conveyor idlers is critical for ensuring safe and efficient operation of industrial conveying systems. However, existing methods often suffer from limited scalability and delayed fault detection, particularly under variable environmental conditions. In this work, we propose MIRA, a transformer-based framework for monitoring idler roller anomalies, which detects and localizes faults using acoustic and vibration signals collected from low-cost sensors. MIRA employs a masked transformer-based autoencoder trained in an unsupervised manner to reconstruct normal patterns and detect deviations via reconstruction error. MIRA can also infer the fault location, enabling spatially aware anomaly detection without the need for labeled data. We validated the system on a custom-built conveyor belt testbed equipped with sensor units, each measuring sound and two-axis vibration signals. We evaluated MIRA on four types of idler faults across 14 roller locations and 6 belt speeds. The results show that MIRA achieves an anomaly detection accuracy of 98.70% and a fault localization accuracy of 96.09%, demonstrating its robustness and practical applicability in complex operational settings.

## 1. Introduction

Belt conveyor systems play a vital role in industrial productivity by transporting bulk materials such as coal, grains, and salts in sectors including mining, agriculture, and manufacturing. However, failures such as belt wear, misalignment, and idler damage can significantly degrade performance and pose serious safety risks. One of the most critical components prone to failure is the idler, which plays an essential role, as it supports the belt load and helps distribute material weight evenly. A malfunctioning idler can lead to excessive friction and belt misalignment, increasing the risk of fire and potentially causing overall system failure. As such, continuous monitoring of the idler condition is essential for maintaining safe and stable conveyor operation.

The traditional method to monitor belt conveyor idler status has mainly relied on manual inspection by an operator. During operation, the operator walks near the running conveyor to visually identify faults or assess them based on vibrations and sounds generated by intentionally striking the idler frame. However, this approach poses significant safety risks, requiring proximity to moving machinery and a considerable amount of time and effort to inspect all idlers due to the large scale of conveyor systems. To address these challenges, various automated idler monitoring techniques have been developed. For instance, numerous studies have utilized vision-based analysis [1,2,3,4], inspection robots [5,6,7], vibration or sound signals [8,9,10], and smart idlers [11] can be used to detect belt conditions or identify idler abnormalities. Commercial solutions such as sensor attachments to the idler [12], fiber optic sensing [13], and a smart idlers [14] have also been introduced. These methods provide more efficient and safer idler monitoring compared to manual inspection.

However, the aforementioned methods have several limitations. They often fail to detect idler faults at an early stage or are restricted to monitoring only a single idler at a specific location. Additionally, some of these methods face challenges when applied in industrial environments, as their performance can be affected by changes in belt speed or environmental factors such as weather and temperature fluctuations. These limitations highlight the necessity of developing more robust and scalable monitoring technologies to ensure continuous and stable operation of the system.

Recently, a number of studies have applied deep neural networks (DNNs) to anomaly detection by analyzing complex patterns in multivariate data and extracting short- and long-term dependencies as features [2,15]. These approaches can also be applied to idler monitoring, particularly for detection of idler anomalies using vibration or sound signals [16,17]. While effective in controlled settings, challenges still remain when deploying them reliably in real-world industrial environments, especially under varying operational and environmental conditions.

To ensure practical deployment of idler monitoring systems, three key challenges must be addressed: (1) scalability, (2) early detection, and (3) low false alarms. First, as a conveyor can range from meters to tens of kilometers [18] in length, the monitoring system must be easily scalable, regardless of its size. Instead of relying on expensive equipment, the system should utilize cost-effective sensors to enhance scalability. Second, early detection is crucial to minimize system downtime and prevent more severe accidents. To achieve this, initial failures must be detected promptly with minimal human intervention, which is essential for both safety and operational efficiency. Lastly, the system must achieve high detection accuracy with minimal false alarms, even in complex environments where various types of idler malfunctions can occur. A high false-alarm rate not only leads to unnecessary maintenance and operational interruptions but also undermines trust in the monitoring system, ultimately lowering its practical utility in real-world applications.

We propose MIRA, an end-to-end idler roller monitoring system that leverages acoustic and vibration sensors to detect anomalies, as well as locate them in real time. To ensure robustness and scalability in large-scale deployments, we adopt a reconstruction-based masked transformer autoencoder model, which learns to reconstruct normal sensor patterns and detects anomalies based on reconstruction errors. The model is trained in an unsupervised manner, allowing it to learn normal operation patterns without requiring labeled fault data, which is often scarce or impractical to obtain in real-world settings. Random masking during training encourages the model to focus on meaningful patterns rather than just memorizing data, thereby improving generalization to unseen anomalies. Furthermore, MIRA is designed to not only detect single-point faults but also infer fault locations across adjacent rollers, enhancing its utility in long conveyor systems. Through extensive experiments on a real-world testbed with diverse operating conditions, we demonstrate that MIRA achieves high accuracy, even under challenging environments such as during rainfall.

**Figure 1:**
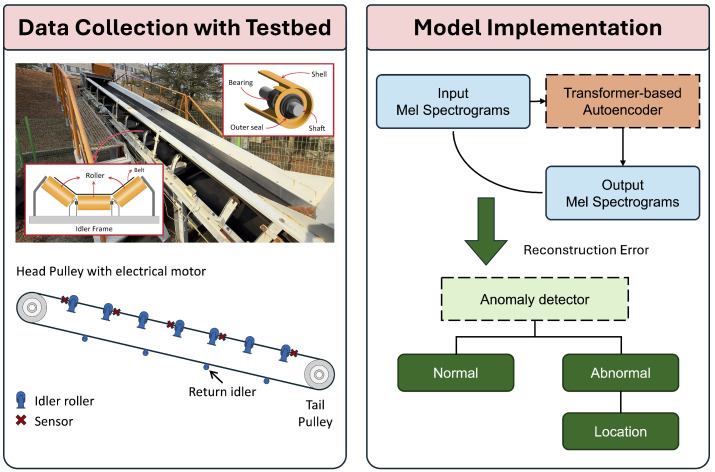
Overall workflow of MIRA.

Our key contributions are outlined as follows. (1) We developed a framework that detects and locates faults across multiple rollers using spatial sensor data. (2) We set up a testbed and installed acoustic and vibration sensors to collect data, which were then used with a masked transformer-based autoencoder for unsupervised anomaly detection. (3) We verified the system’s practicality through experiments under various fault types, belt speeds, and environmental conditions. Figure 1 illustrates the end-to-end workflow of MIRA.

The rest of this paper is organized as follows. Section 2 reviews the background of idler roller faults and related works. Section 3 outlines the overall design and implementation of MIRA. Section 4 presents experimental results, and we discuss the implications and limitations in Section 5 before concluding the paper.

## 2. Related Works

This section introduces the operational context and failure types of idler rollers in belt conveyor systems. It also reviews existing monitoring methods, such as vision-based inspection, robotic systems, acoustic sensing, and deep learning approaches. While these methods show promise, they often face challenges in generalization and robustness under real-world conditions.

### 2.1. Idler Rollers Their Faults in Belt Conveyor Systems

**Figure 2:**
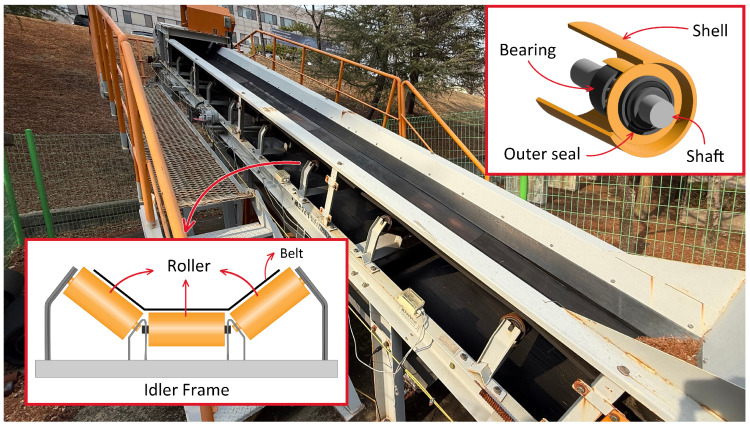
Conveyor belt testbed and idler structure.

The idler roller plays a crucial role in a belt conveyor system, facilitating the movement of bulk materials to other locations. To collect data for idler fault detection, we constructed a 7 m conveyor belt testbed, as illustrated in Figure 2. A commonly used idler configuration is a trough-shaped frame, which consists of three rollers to support the belt. This structure enhances the load-carrying capacity and provides stable support to prevent spillage [19].

Nevertheless, idler faults can arise from various causes, including belt misalignment, excessive loads, harsh environmental conditions, and lack of maintenance [20]. For instance, the improper installation of the idler may lead to shaft deflection, resulting in the idler becoming stuck or detaching due to imbalance. Excessive loads can induce mechanical stress on the idler components, which can accelerate wear or even lead to structural failures over time. Additionally, external environmental factors such as high temperature and exposure to moisture can accelerate idler failures. These factors include joint failures caused by mechanical stress or environmental degradation, as well as maintenance-related issues such as over-greasing or insufficient lubrication, all of which can lead to tearing or separation at the joints and significantly reduce the lifespan of both the idler and the bearing [21].

### 2.2. Belt Conveyor Monitoring Methods

#### 2.2.1. Non-Acoustically Based Monitoring

Various studies have proposed automated belt conveyor monitoring, including vision-based analysis, robotic systems, and smart idlers. Most of them monitor the overall status of the conveyor belt, identifying phenomena such as tearing and damage to the belt, as well as detecting idler faults.

There are several vision-based approaches for fault detection that leverage machine learning [1,2,3,4]. Siami et al. [1] used infrared images to detect overheated idlers in belt conveyor systems. Liu et al. [2] developed an end-to-end model that integrates temporal and spatial features using temporal convolutional networks for detection of belt damage. Wang et al. [3] applied the YOLOv7 model to identify instances of longitudinal tearing while optimizing speed and accuracy through model scaling based on detection results. However, vision-based methods face several limitations when applied to idler monitoring, such as the need to enhance robustness and generalization under low-light or poor-quality imaging conditions, as well as difficulty handling unseen idlers, which often results in degraded performance.

Recent studies have explored the use of mobile unmanned ground vehicles for inspection of belt conveyors in hazardous environments. Robotic systems utilize a variety of onboard sensors for real-time fault detection [5,6,7]. Cao et al. [5] developed a suspension inspection robot that uses infrared thermal imaging and gas sensors to monitor belt conditions. Dabek et al. [7] proposed a method to detect overheated idlers by combining RGB and infrared images collected by robots. However, deploying robots in real-world conveyor systems remains challenging due to compatibility issues with diverse conveyor structures and the requirement for extra space to operate safely within constrained environments.

Smart idlers with embedded sensors have been developed to monitor the condition of idler rollers. These methods utilize energy-harvesting devices, allowing for autonomous operation without reliance on external power sources. Zhou et al. [11] designed an embedded belt conveyor idler monitoring node with an energy management circuit that enables real-time tracking of idler temperature and vibration. Additionally, recent studies have proposed sensing idlers based on fully enclosed bearing-structured self-powered rotation sensors [22]. Despite these advancements, practical application of these newly designed systems requires significant installation costs and advanced sensors to enhance real-time data acquisition.

#### 2.2.2. Acoustically Based Monitoring

**Figure 3:**
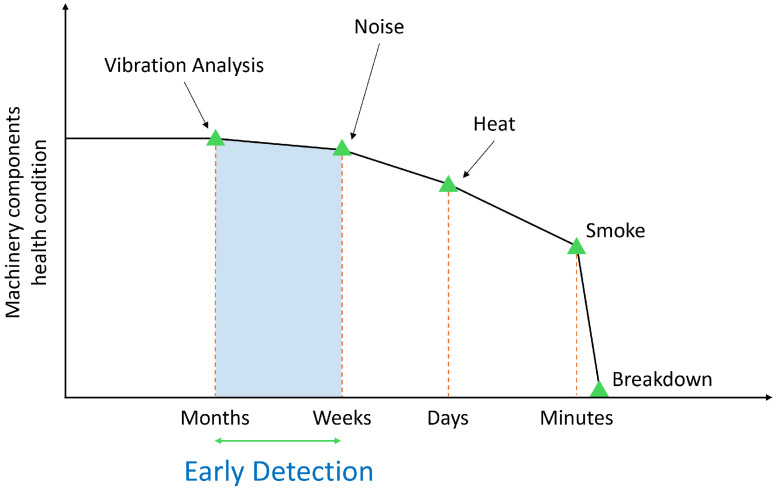
Theoretical P-F curve showing that vibration and acoustic signals enable earlier detection than thermal indicators.

Sound and vibration sensorscan also be utilized to detect idler faults, as they have broad applicability and low installation cost. Figure 3 illustrates the theoretical P-F (Potential–Failure) curve, which indicates that vibration and acoustic signals manifest significantly earlier than thermal anomalies [23,24]. Specialized equipment such as distributed acoustic sensing (DAS) devices is employed to detect idler faults using sound [13], but their high cost makes their use challenging for broad installations. Hence, research has focused on detecting idler faults efficiently using sound or vibration.

Peng et al. [8] utilized audio signals to detect idler faults through wavelet packet transformation and a convolutional neural network (CNN). The proposed method enabled easier classification of data features for fault diagnostics. Zhang et al. [9] effectively identified idler failures using multi-frame fusion optimization of Mel-frequency cepstral coefficient (MFCC) features and a weighted support vector data description model. This approach complements the noise vulnerability of conventional methods. Ban et al. [10] proposed an optimized model capable of reliably detecting idler faults, even in high-noise environments. This method also reduced the computational load compared to traditional approaches. Alharbi et al. [25] enhanced YAMNet, enabling temporal feature extraction and optimized identification processes, leading to better idler fault classification performance.

### 2.3. Transformer-Based Deep Learning for Time Series

#### 2.3.1. Transformers in Multivariate Analysis

Transformers use multi-head attention and account for diverse representations to capture periodic patterns in time-series signals effectively. Transformer-based frameworks in multivariate time series have been effectively applied to various tasks, such as classification, forecasting, and anomaly detection [26]. TARNet [27] enhanced end-task performance through task-aware data reconstruction. Gorbett et al. [28] showed that sparse and binary-weighted transformers could achieve favorable performance comparable to that of dense floating-point models while reducing computational complexity.

#### 2.3.2. Transformer-Based Anomaly Detection

Numerous studies have applied transformers to anomaly detection. TransAnomaly [29] advanced an unsupervised anomaly detection algorithm, combining VAE and transformers. Chen et al. [15] proposed graph learning with a transformer for anomaly detection that automatically learns temporal dependencies using a multi-branch attention mechanism. Xu et al. [30] introduced a new anomaly attention mechanism to measure association discrepancy for unsupervised time-series anomaly detection. Additionally, DCT-GAN [31], TranAD [32], and CDAM [33] enhance generalization and temporal trend learning and address concept drift adaptation, respectively. Meanwhile, CAT [34] and MEMTO [35] address scalability and training stability in anomaly detection tasks.

Despite these advancements, most existing transformer-based anomaly detection methods focus primarily on capturing temporal dependencies within a single time series, often treating multivariate channels as abstract feature vectors, without considering their physical and spatial arrangement. Therefore, these methods generally lack the ability to spatially localize faults across distributed infrastructure. In contrast, MIRA is specifically designed to exploit spatial correlations in distributed sensor arrays. MIRA also utilizes random masking in training to reconstruct missing sensor patches based on the context provided by neighboring sensors. This makes the model capable of learning robust inter-sensor dependencies rather than simply memorizing temporal patterns.

## 3. Materials and Methods

This section presents the overall design and implementation details of MIRA, a framework designed for idler roller anomaly detection and fault localization. It covers the dataset, model architecture, and inference strategy.

### 3.1. Data Collection

**Figure 4:**
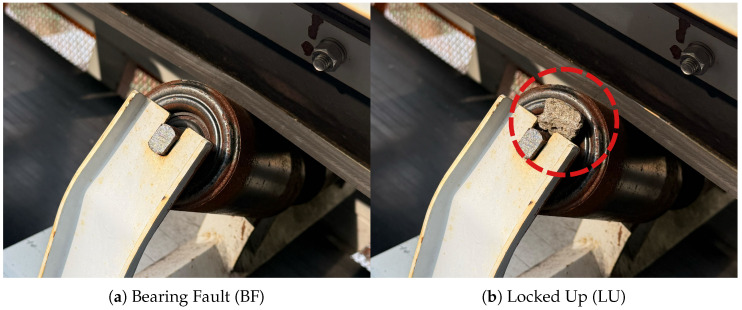

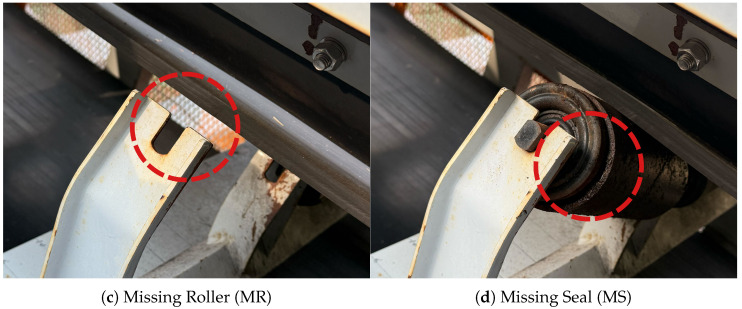
Four types of idler roller faults considered in the experiments.

**Figure 5:**
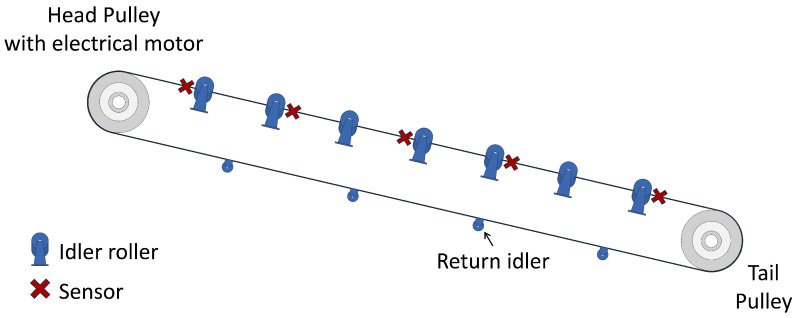
Sensor and idler roller layout in the testbed. Only one side is shown for clarity, as the arrangement is symmetrical.

We conducted the experiment on 14 roller locations, inducing faults at each position. The fault types are detailed in Figure 4. For each roller location, 4 fault scenarios were simulated: (a) Bearing Fault (BF)—foreign matter such as dirt, dust, and metal swarf was introduced into one side of the roller bearing to artificially contaminate it, hindering its smooth movement; (b) Locked Up (LU)—a small stone was inserted between the roller and idler frame so that the roller could not rotate; (c) Missing Roller (MR)—the roller was removed from the idler frame; (d) Missing Seal (MS)—one side of the roller was operated with a detached outer seal. Figure 5 illustrates the layout of sensors and idlers on the testbed, arranged symmetrically on both sides. A total of 10 sensor units were evenly spaced along the conveyor’s side frame, ensuring no interference with the operation of the idlers or a belt. The return idler and the middle roller of the carrying idler were excluded from the experiment due to the difficulty of detaching the rollers from the frame.

#### 3.1.1. Hardware Setup

**Figure 6:**
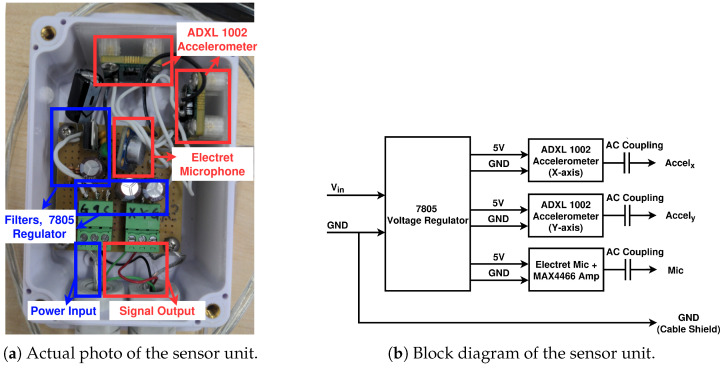
Implementation of the sensor unit.

A total of 10 sensor units were built and connected to an USB-6353 DAQ (from National Instruments, Austin, TX, USA) unit to collect acoustic and vibrational samples of the belt conveyor. The DAQ unit was connected to an edge PC, which was remotely controlled over Wi-Fi to collect and process the samples. Each sensor unit was enclosed in a weatherproof enclosure, consisting of two ADXL 1002 accelerometers (from Analog Devices, Wilmington, MA, USA) and a CMA-4544PF-W electret microphone (from Same Sky, Lake Oswego, OR, USA) with a MAX4466 amplifier (from Analog Devices, Wilmington, MA, USA), as shown in Figure 6. DC voltage was supplied to the sensor unit and stepped down to 5 V by an internal LM7805 regulator (from Texas Instruments, Dallas, TX, USA). The resulting DC voltage was then filtered and supplied to the accelerometers and the microphone as needed. The accelerometers and microphone output analog signals, which were high pass-filtered for DC offset removal, then sent to the DAQ unit via a shielded wire for digitization. All parts were imported to Pohang, South Korea by local suppliers.

The DAQ unit is a multiplexed 32-channel DAQ with maximum sampling rate of 1 MS/s [36]. It was used to digitize the analog outputs of our sensor units coherently and synchronously. The obtained samples were then transferred to an edge PC and later collected and transferred into remote workstations for further processing.

The typical current draws of an ADXL1002 accelerometer and the microphone module used in this work are 1.0 mA and 26 μA, respectively [37,38]. This means that the amount of power required by our sensor unit was approximately 10 mW, and powering these sensor units with battery or solar cells would be possible if we replaced the DAQ and the edge PC with low-power ADCs and MCUs. Furthermore, the sensor units can be duty-cycled to reduce the power budget further, to the point where they can last for years with a single-cell battery if needed.

Data samples were collected from the DAQ unit with a Python script built upon the manufacturer’s official Python API [39]. The collected samples were then either dumped into files to create training datasets or streamed to other processes via TCP. To collect the samples, the script was configured to continuously scan the 32 analog channels of the DAQ unit at a sampling rate of 31.25 kS/s per channel (the maximum achievable per channel sampling rate of this particular DAQ). This is higher than the Nyquist rate of the accelerometer units in use [37], which is around 22 kS/s (11 kHz), and marginally lower than the Nyquist rate of the microphone units in use [40], which is around 40 kS/s (20 kHz). In practice, this is not a major problem, as most of the observed frequency components are far below the Nyquist rate of the sensors. Only 30 channels of the DAQ unit were actually connected to the sensors.

To create the datasets, the collected data samples were dumped into files. The SigMF standard [41] was used to format and store the data samples, as it is a well-specified way of storing long, multivariate time-series data samples in files, ensuring the portability of the recorded data files. These files were then fetched and moved to workstations over Remote Desktop Protocol.

#### 3.1.2. Dataset Overview

Table 1 lists two datasets: $D_{1}$, the main dataset, contains normal and abnormal data collected over a 2-month period, while $D_{2}$ includes data collected while raining. The conveyor belt speed was controlled by adjusting the motor’s inverter frequency, which ranged from 0 to 60 Hz, and the experiment was conducted at six speed levels (10–60 Hz in 10 Hz increments). Data collection was distributed across multiple days rather than consecutively recording. For instance, 10 h of normal $D_{1}$ data at 60 Hz was obtained over five non-consecutive days, with 2 h sessions per day. Abnormal data was collected in 20 min sessions for every combination of 14 roller locations, 4 fault types, and 6 belt speeds, accumulating a total of 112 h of data. Normal data was split into train/validation/test sets in a 6:2:2 ratio. As the approach follows an unsupervised learning model, all abnormal data was used exclusively for inference.

Each sensor unit consists of one acoustic sensor and two vibration sensors that measure vibrations along the *x* and *y* axes, respectively. This configuration results in total 30 channels across 10 sensors that are all connected to an NI USB-6353 DAQ and sampled at 31.25 kS/s.

### 3.2. MIRA Architecture

**Figure 7:**
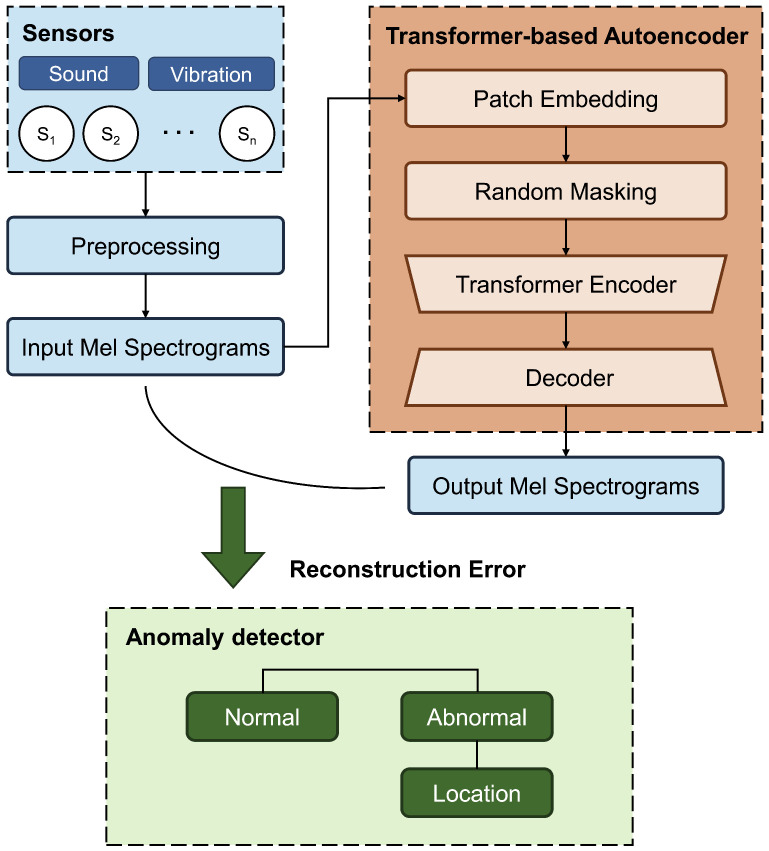
Overall architecture of MIRA.

Figure 7 depicts the overall architecture of MIRA. The system comprises *N* sensors, each with three channels, leading to a total of 3N sensor streams that undergo preprocessing. First, a fifth-order Butterworth high-pass filter with a 300 Hz cutoff frequency was applied to remove DC components. This cutoff frequency was empirically chosen to filter out low-frequency noise caused by wind; rain; and vibrations that are irrelevant to idler fault detection, such as motor rotation or belt flapping. A 2 s sliding window was applied to the sensor streams to achieve a frequency resolution of approximately 0.5 Hz. This duration was empirically selected to capture fine-grained spectral sidebands associated with idler faults. A 50% overlap was used during training, while non-overlapping windows were used for validation. The resulting segments were then transformed into normalized Log-Mel spectrograms.

The preprocessed sensor streams were fed into the transformer-based autoencoder, which basically reconstructed the input. We used a transformer for the encoder, as it captures global context more effectively than CNNs and its self-attention mechanism helps model the relationships between sensors. To prevent the model from just memorizing normal data patterns and ensure it instead learned meaningful representations, we applied random zero masking to the patches, introducing a regularization effect. The decoder uses two stacked transposed convolutions to reconstruct input from latent representations. Section 3.3 details the transformer-based autoencoder.

After obtaining reconstructed input, we calculated the reconstruction error by computing the mean squared error (MSE) between the input and output Mel spectrograms for each sensor channel. If the error for the sensor exceeded a certain threshold, the input was classified as abnormal. By leveraging the spatial layout of the sensors along the testbed, the system then estimates the faulty location by identifying the group of rollers located nearest to the abnormal sensors. The detailed mechanism for the anomaly detector is further described in Section 3.4.

### 3.3. Transformer-Based Autoencoder

#### 3.3.1. Patch Embedding and Positional Encoding

As illustrated in Figure 7, the model processes the input Mel spectrogram ($X\in R^{C\times H\times W}$) as a sequence of patch embeddings. Here, *C* represents the number of sensor channels (30), *H* is the number of frequency bins (128), and *W* is the number of time steps (62) derived from the 2 s window. To extract meaningful representations from the input, employed 2D convolution with a kernel size of 4 and a stride of 4, followed by batch normalization and ReLU activation. To preserve spatial information critical for identifying where the fault occurred, learnable 2D positional encodings ($E_{pos}$) were added element-wise to the patch embeddings. The final input sequence (*Z*) is defined as follows:

(1) $$Z=ReLu\left(BatchNorm\left(Conv2D\left(X\right)\right)\right)+E_{pos}$$

#### 3.3.2. Random Masking and Encoder

To learn robust feature representations in an unsupervised manner, we applied a random patch masking before encoding the input. A random binary mask (*M*) was generated and applied patch-wise ($Z_{masked}=Z⊙M$), removing 20% of the patches. This forces model to rely on the spatial and temporal correlations present in the visible patches to infer the missing context.

Since transformers operate on sequences, we flattened $Z_{masked}$ into a tokenized representation; then, these tokes were processed by the transformer encoder, which is composed of 6 stacked layers. Each layer contains 8 self-attention heads and a feed-forward network with a dimension of 1024 and a dropout rate of 0.1. This step enables the model to generalize by reconstructing missing information and to capture dependencies across the channels, which is crucial for identification of anomaly locations.

#### 3.3.3. Decoder and Reconstruction

The encoded latent representation was reconstructed in the original Mel spectrogram space through a two-layer transposed convolutional layer with a kernel size of 4, stride of 2, and padding of 1. This decoder progressively upsamples the spatial dimensions while reducing the channel dimension back to the input size. To ensure exact alignment with the input resolution (H,W), a bilinear interpolation was applied. The model was trained to minimize the MSE lossbetween the original spectrogram (*X*) and the reconstruction ($\hat{X}$).

Crucially, the loss is computed only in the masked regions. This self-supervised objective is important, as it maximizes the information density of the encoder’s representation by forcing it to predict the hidden content based solely on the context of neighboring, unmasked sensors. The reconstruction loss is calculated as follows:

(2) $$L=\frac{\sum ({\left(X-\hat{X}\right)}^{2}⊙\left(1-M\right))}{\sum (1-M)}$$

where (1−M) selects the indices of the original patches that were masked. This reconstruction step expects that normal inputs are reconstructed accurately, while anomalous inputs lead to higher reconstruction errors, which can be used for the next step.

### 3.4. Anomaly Detector

To determine whether a test sample indicates an abnormal condition, we computed the reconstruction error between the input and the output Mel spectrograms using the MSE. For each sensor unit, which includes one acoustic and two vibration channels, the MSE is calculated independently for each channel. The reconstruction error of the sensor is then defined as the average of the three channel-wise errors.

To establish detection thresholds, we performed inference on all samples from the normal validation set using the trained model. For each 2 s window, we computed reconstruction errors across all sensors. The threshold for each sensor was then determined by selecting the maximum reconstruction error observed in the validation set. This ensures that the threshold reflects the worst-case scenario under normal conditions for that specific sensor, allowing for identification of the unique characteristics and noise profiles of individual sensor locations.

During testing, if the reconstruction error of the sensor exceeds its threshold, the sensor is flagged as abnormal. To identify the likely location of the fault, we leveraged the physical sensor-to-roller mapping depicted in Figure 5. Specifically, sensors located at the top-most and bottom-most positions of the testbed are associated with their two adjacent rollers, while all other sensors are associated with three neighboring rollers. If any associated sensor detects an anomaly, we flag the nearby rollers as potentially faulty. This spatial inference enables MIRA to not only detect anomalies but also to localize them effectively along the conveyor belt.

### 3.5. Model Implementation

We implemented MIRA using Pytorch Lightning 2.5.0 with Python 3.11. The transformer encoder consists of six layers, each with 8 attention heads, an embedding dimension of 256, and a feedforward dimension of 1024. Dropout with a rate of 0.1 was applied to prevent overfitting. Input Mel spectrograms were patch-embedded and subjected to random masking, with 20% of the patches masked during training. The effect of varying masking ratios on model performance is analyzed in Section 4.4.

We used the Adam optimizer with a learning rate of 1 × 10^−4^ and a batch size of 256. The model was trained to minimize the MSE between the input and reconstructed Mel spectrograms, with the loss computed only on the masked patches. Early stopping was applied based on the validation loss, with patience set to 10 epochs. Although the number of training epochs varied depending on the dataset and training conditions, most runs converged around 300 epochs, with each epoch taking approximately 1 min. All experiments were conducted on a server equipped with two NVIDIA A100 GPUs.

## 4. Results

This section evaluates the performance of MIRA under diverse operating conditions. Detection and localization accuracy are analyzed across various fault types and belt speeds.

### 4.1. Evaluation Metric

We evaluate MIRA on two tasks: anomaly detection and fault localization based on detected sensor anomalies.

(1) Anomaly detection accuracy: We compute the classification accuracy of detecting whether each 2 s segment is normal or abnormal. This is evaluated across five categories—normal and four types of faults: BF, LU, MR, and MS. The accuracy is computed as the proportion of correctly predicted labels over the total number of test samples.

(2) Fault localization accuracy: When the sensor is classified as abnormal, we infer the faulty roller location based on its spatial mapping to adjacent rollers. As defined in Section 3.4, the top-most and bottom-most sensors are associated with two adjacent rollers, while all others are linked to three neighboring rollers. Localization estimation is considered correct if at least one of the actual faulty rollers is included in this inferred set of sensors. We report the localization accuracy as the percentage of correctly identified fault locations over the total number of abnormal test samples.

### 4.2. MIRA Performance

To explore the overall detection and localization accuracy of MIRA, we evaluated the performance using the dataset $D_{1}$ in Table 1. For training of the model, we use a total of 18 h of normal samples with speeds of 20, 40, and 60 Hz. For inference, we use 12 h of normal samples and 112 h of abnormal samples for all belt speeds.

Table 2a presents the anomaly detection accuracy across different fault types and belt speeds. MIRA achieved an average accuracy of 97.23% for normal data and near-perfect accuracy for BF, LU, and MS. For the MR, the accuracy was slightly lower, at 95.99%. The slightly lower accuracy for MR may be attributed to the absence of a roller, resulting in a lack of noise features typically present in other faults, making detection more challenging.

Table 2b shows the fault localization accuracy. Overall, MIRA accurately identified fault locations, with an average localization accuracy of 96.09%. In particular, for BF and LU, the localization accuracy slightly decreased at unseen speeds, such as 10, 30, and 50. This suggests that the spatial inference is more sensitive to discrepancies between training and test conditions. Since localization relies on the mapping of spatial patterns of sensor responses, it benefits more directly from exposure to similar distributions during training.

### 4.3. Effect of Speed Variation in Training Data

As demonstrated in the previous section, the performance of fault localization is subjected to the distribution of the normal training data. To analyze the effect of speed variation in the training set, we trained the model under four different combinations of belt speeds in the normal data: (1) all speeds from 10 to 60 (denoted as 10–60), (2) three sparsely sampled speeds of 10/30/50, (3) another set of three speeds of 20/40/60, and (4) a single-speed setting using only 60. For a fair comparison, the first three configurations each used 18 h of data, while the 60-only model was trained with 8 h of data after excluding 2 h reserved for testing.

**Figure 8:**
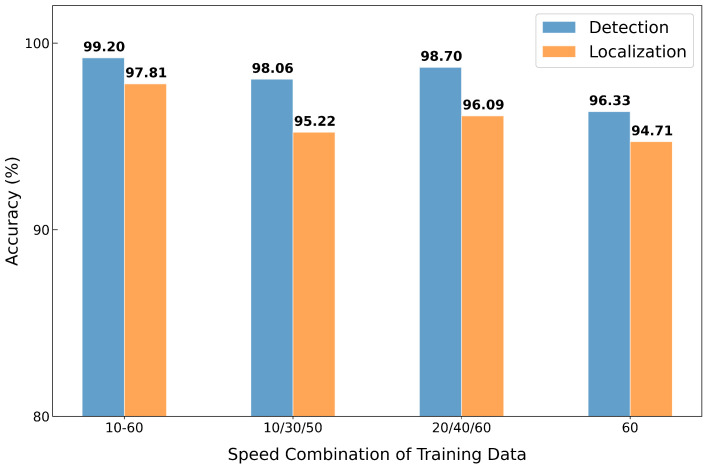
Anomaly detection and fault localization accuracy of MIRA with varying combinations of belt speeds used in training.

Figure 8 shows the anomaly detection and localization accuracy under each combination. As expected, the model trained with the full range of speeds achieved the highest accuracy on both tasks, as it covered all speeds in the test set. The 60-only model showed the lowest performance but still achieved around 95% accuracy, which highlights the model’s ability to generalize to unseen conditions. The models trained with three speed combinations showed similar performance, with the 20/40/60 combination performing slightly better.

While training with all speeds yielded the best results, such training is often impractical in real-world deployments, as we cannot train models for every condition. We therefore used 20/40/60 for the remaining experiments, as this combination provided comparable results.

### 4.4. Effect of Masking Ratio

**Figure 9:**
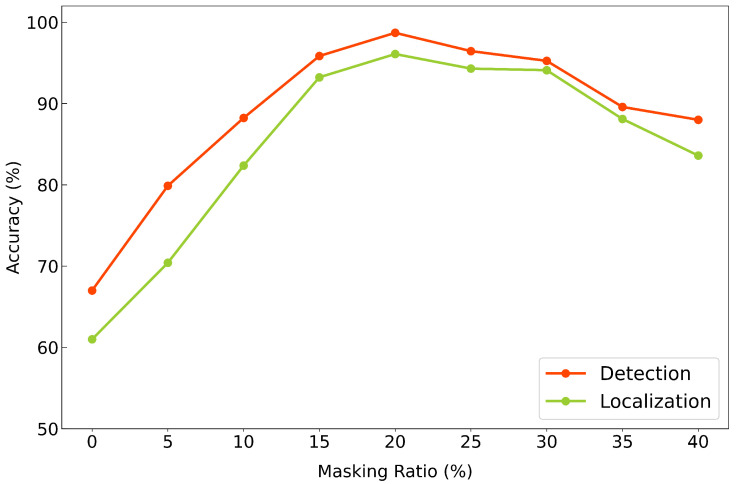
Effect of masking ratio on anomaly detection and fault localization accuracy.

Figure 9 illustrates the anomaly detection and fault localization accuracy across various masking ratios, ranging from 0% to 40% in 5% increments. When no masking was applied before the transformer encoder, the model showed significantly low accuracy on both tasks, likely due to overfitting to the training set and poor generalization. On the other hand, at a high masking ratio of 40%, the model struggled to reconstruct the input effectively, leading to decreased performance, presumably due to the excessive loss of information during training.

Among all configurations, a masking ratio of 20% achieved the highest performance on both tasks. This result suggests that 20% serves as the optimal point of balance, where the model is sufficiently regularized to generalize well while retaining enough information to reconstruct meaningful features.

### 4.5. Robustness to Environmental Changes

To evaluate the generalization capability of MIRA under unseen environmental conditions, we evaluated the model trained exclusively on normal data from $D_{1}$ using the test set from $D_{2}$ in Table 1, which was collected during rainfall. As shown in Table 3, the MS maintained high accuracy in both anomaly detection and localization, even under rainy conditions. However, for normal and BF accuracy, performance notably decreased, especially in the localization accuracy of BF.

We further examined whether retraining with additional data from the rainy environment could improve performance. After augmenting the training set with normal rainy-condition samples from $D_{2}$, we observed improved accuracy on both tasks. This result indicates that while MIRA is robust to mild environmental changes (e.g., changes in belt speed), larger variations such as changes in weather conditions can degrade performance. Incorporating adaptive techniques such as online learning may help the system maintain high reliability across diverse environments.

### 4.6. Scalability with Varying Numbers of Sensors

MIRA is flexible in terms of the number of sensors, making it suitable for environments where the ability to install sensors may be limited due to cost or physical constraints. While our testbed utilized 10 sensor units, we evaluated the system’s robustness under a reduced sensor countto assess scalability performance. Starting from the original 10-sensor setup shown in Figure 5, we progressively removed sensors in the following order: the top-most two sensors, then the bottom-most two and the two central sensors. In the default configuration, each sensor is associated with three neighboring rollers for fault localization, except the top-most and bottom-most sensors. However, under the four-sensor condition, this mapping was revised so that each sensor covered four rollers to ensure full coverage of the fourteen rollers.

**Figure 10:**
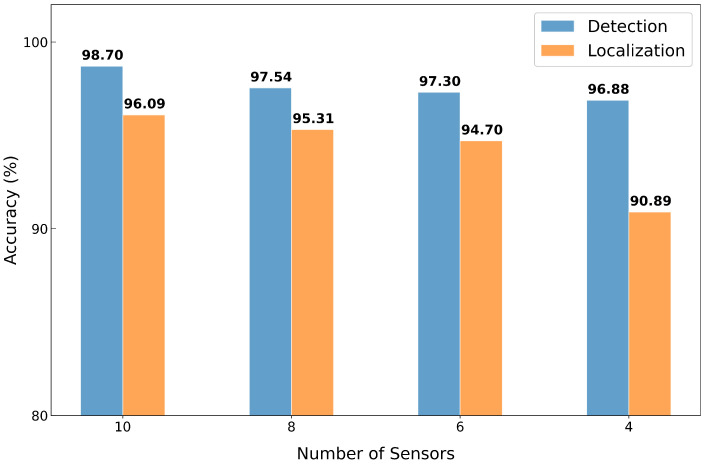
Detection and localization accuracy of MIRA with varying numbers of sensors deployed on the testbed.

As shown in Figure 10, performance on both tasks decreases as the number of sensors is reduced. However, detection accuracy remains relatively stable, even with fewer sensors. This is because the anomaly detection threshold is determined based on the worst-case reconstruction error observed in the normal validation set, making it less sensitive to sensor reduction. On the other hand, fault localization accuracy declines more noticeably, given that fewer sensors provide coarser spatial resolution. Nevertheless, MIRA still achieves 90.89% localization accuracy with only four sensors, demonstrating its robustness and adaptability for scalable deployments.

## 5. Discussion

This study demonstrated that the MIRA framework can effectively detect and localize idler roller anomalies in a belt conveyor. This section provides an in-depth discussion of MIRA’s real-time applicability, its robustness to environmental changes, the limitations of the current model, and its future generalizability.

### 5.1. Inference Speed and Real-Time Applicability

To verify MIRA’s applicability in real industrial settings, we evaluated its runtime performance. Based on a 2 s, 30-channel sensor data segment, preprocessing required an average of 182.93 ms, while inference by the masked transformer autoencoder took an average of 3.18 ms. The total latency is approximately 186.11 ms, which is significantly shorter than the 2 s data acquisition window. This suggests that MIRA is capable not only of simple real-time monitoring but also of providing near-instantaneous feedback. This high speed is a result of the balance struck between performance and speed, achieved through the efficient architecture of the transformer encoder and the lightweight transposed convolution decoder. Such rapid inference not only allows for immediate alarms upon fault detection but also serves as a foundation for more advanced monitoring systems, such as the tracking of fault progression or performing re-analysis across multiple segments.

### 5.2. Robustness to Environmental Changes and Adaptability

Industrial sites are often exposed to unpredictable environmental changes. In this study, we tested MIRA’s performance under an uncontrolled environmental variable—rainfall. As shown in Table 3, the rainy condition affected the model’s performance, particularly the localization accuracy for BF faults. A noteworthy point is that this performance degradation was recovered to 90.72% by retraining the model with additional normal data collected during the rainfall. This demonstrates MIRA’s potential to adapt to new environments. This adaptation likely occurs because the acoustic and vibration signals from rain add a consistent, broadband noise signature across all sensor channels. As the model learns this new normal state, the autoencoder learns to effectively reconstruct and ignore environmental noise patterns. Consequently, the subtle, unique anomaly patterns of the BF fault, which were previously obscured by the rain noise, become more clearly exposed as reconstruction errors, resulting in improved detection and localization accuracy.

This result indicates that, for practical field deployment, strategies like online learning or incremental learning to periodically retrain the model on new normal data will be crucial for maintaining the system’s long-term robustness and reliability. To realize such a robust field deployment, we will utilize an incremental online learning strategy focused on autonomous adaptation. This involves maintaining a sliding normal data buffer of sensor data and periodically applying a low learning rate for fine-tuning. This approach enables the model to subtly adjust its baseline representation of normal to accommodate environmental drift.

### 5.3. Analysis of Localization Performance and Limitations

Although MIRA achieved a high average localization accuracy of 96.09%, it exhibited performance variations under specific conditions, as shown in Table 2b. Notably, the LU fault recorded relatively lower accuracy at speeds not used during training. This suggests that the signal characteristics of the LU fault at these speeds may differ from the patterns at the trained speeds. A possible explanation is that at certain untrained low or high speeds, the vibration and acoustic signals from the LU fault may not propagate strongly to the nearest sensor but, instead, tend to diffuse weakly across a wider area of sensors.

MIRA’s current localization method relies on whether an individual sensor’s reconstruction error exceeds a threshold and on a predefined heuristic of physical sensor-to-roller proximity. If an anomalous signal is weak and widely diffused, it may not cross the clear threshold at the nearest sensor, leading to localization failure or inaccuracy. This is a clear limitation of the current approach, and future research should aim to improve it. For example, a secondary classifier or a graph neural network could be introduced, taking the entire reconstruction error pattern from all sensors as input. This would allow the model to learn a unique spatial error fingerprint corresponding to specific fault types and locations. Such an approach is expected to provide improved localization robustness for ambiguous or diffused fault signals compared to the current heuristic-based method.

### 5.4. Generalizability and Future Applications

Although this study focused on single-point failures for controlled validation, MIRA’s architectural design is not inherently limited to detecting only single faults. The autoencoder processes all sensor channels simultaneously, and the anomaly threshold is applied independently to each sensor unit. Therefore, the system is, in principle, capable of detecting multiple non-adjacent, simultaneous faults.

Furthermore, the core principle of MIRA, a masked autoencoder learning spatio-temporal normal patterns from distributed low-cost sensors, has high generalizability. Beyond conveyor belt systems, it can be applied to various other fields, such as structural health monitoring for bridges, fault localization on linear railway tracks, or condition monitoring for machinery with multiple connected rotating parts, like escalators or wind turbines. The key requirement is the existence of a distributed sensor network from which normal operational data can be easily collected.

## 6. Conclusions

We presented MIRA, a transformer-based framework for detecting and localizing anomalies in belt conveyor idler rollers. MIRA leverages acoustic and vibration signals collected from multiple spatially distributed sensors and can effectively identify abnormal patterns without requiring labeled fault data. The system is both robust and scalable, capable of generalizing across varying operational conditions and sensor configurations. To validate its effectiveness, we deployed custom-built sensors on a real-world conveyor belt testbed and demonstrated high detection and localization accuracy across diverse fault types and environmental scenarios.

While MIRA showed strong performance, we acknowledge that testing under more severe industrial environments was not simulated due to experimental constraints. Our immediate future work will therefore focus on full-scale operational deployment to validate scalability under real-world environmental changes, refining proposed online learning strategies for sustained autonomous adaptation and reliable operation.

## Figures and Tables

**Table 1:** Summary of datasets.

Dataset	Condition	Abnormal Type	# of Abnormal Locations	Belt Speed (Hz)	Total Duration (h)
$D_{1}$	Normal	-	-	10–60 (step 10)	60
	Abnormal	BF, LU, MR, MS	14	10–60 (step 10)	112
$D_{2}$ (rain)	Normal	-	-	20, 40, 60	6
	Abnormal	BF, MS	3	20, 40, 60	6

**Table 2:** Overall detection and localization performance of MIRA (in % accuracy) under various belt speeds and fault types. The model was trained using only normal data collected at 20, 40, and 60 Hz. (**a**) Anomaly detection accuracy for each fault type at various belt speeds; (**b**) fault localization accuracy at various belt speeds.

(**a**)					
**Speed (Hz)**	**Normal Acc.**	**BF Acc.**	**LU Acc.**	**MR Acc.**	**MS Acc.**
10	97.81	100	99.73	91.25	96.26
20	99.00	100	100	96.23	100
30	95.11	100	100	98.35	100
40	96.22	100	100	94.57	100
50	96.89	100	100	99.94	100
60	98.36	100	100	95.60	99.99
Average	97.23	100	99.95	95.99	99.38
(**b**)					
**Speed (Hz)**	**BF Acc.**	**LU Acc.**	**MR Acc.**	**MS Acc.**	
10	93.55	85.90	99.52	92.20	
20	99.99	98.32	95.38	99.95	
30	96.46	87.39	95.87	99.95	
40	100	99.52	98.01	99.98	
50	90.42	80.38	97.76	99.90	
60	99.08	96.89	99.83	100	
Average	96.58	91.40	97.73	98.67	

**Table 3:** Detection and localization accuracy under rainy conditions before and after adding normal rainy-condition data (* after model update). (**a**) Anomaly detection accuracy in rainy conditions; (**b**) fault localization accuracy in rainy conditions.

(**a**)					
**Speed (Hz)**	**Normal Acc.**	**BF Acc.**	**MS Acc.**	BF * Acc.	MS * Acc.
20	90.97	93.33	100	98.67	100
40	97.56	95.50	100	99.18	100
60	91.86	95.39	100	100	100
Average	90.13	94.74	100	98.28	100
(**b**)					
**Speed (Hz)**	**BF Acc.**	**MS Acc.**	BF * Acc.	MS * Acc.	
20	70.24	92.78	88.18	99.94	
40	68.28	99.78	89.69	100	
60	76.33	100	94.28	100	
Average	71.62	97.52	90.72	99.98	

## Data Availability

The datasets presented in this article are not readily available due to an another ongoing study with overlaps in the dataset. Requests to access the datasets should be directed to the authors of the article.
